# *Granulicatella adiacens* and Early-Onset Sepsis in Neonate

**DOI:** 10.3201/eid1710.101967

**Published:** 2011-10

**Authors:** Matthew J. Bizzarro, Deborah A. Callan, Patricia A. Farrel, Louise-Marie Dembry, Patrick G. Gallagher

**Affiliations:** Yale University School of Medicine, New Haven, Connecticut, USA (M.J. Bizzarro, L.-M. Dembry, P.G. Gallagher);; Yale–New Haven Hospital, New Haven (M.J. Bizzarro, D.A. Callan, P.A. Farrel, L.-M. Dembry, P.G. Gallagher)

**Keywords:** bacteria, infants, neonate, sepsis, streptococci, Granulicatella adiacens, granulicatella, letter

**To the Editor:** *Granulicatella* and *Abiotrophia,* formerly known as nutritionally variant streptococci, are normal flora of the human upper respiratory, gastrointestinal, and urogenital tracts (*1*). *G. adiacens* has been associated with bacteremia and endovascular, central nervous system, ocular, oral, bone and joint, and genitourinary infections (*1**–**4*).

Although streptococci are a frequent cause of early-onset sepsis in newborns, non–group B or D streptococci comprise only ≈1% of cases of early-onset neonatal sepsis; the condition is primarily associated with viridans streptococci (*5*). This report describes a male infant with early-onset sepsis caused by *G. adiacens.* Molecular genetic studies identified the same organism in flora isolated from the maternal cervix, which suggests vertical transmission.

After 36 weeks’ gestation, a male infant, weighing 2,850 g, was born by repeat caesarean section to a 37-year-old woman who was negative for group B streptococcus; she began labor without rupture of membranes. Apgar scores were 9 and 9 at 1 and 5 minutes, respectively. Respiratory distress developed in the infant within an hour of birth. Peripheral blood and cerebrospinal fluid (CSF) samples were obtained, and intravenous ampicillin (150 mg/kg every 12 h) and gentamicin (4 mg/kg every 24 h) were administered. Leukocyte count was 27,000/mm^3^ with 79% polymorphonuclear leukocytes and 2% band forms; platelet count was 223,000/mm^3^. CSF cell counts were 3 leukocytes/mm^3^ and 18 erythrocytes/mm^3^.

Respiratory distress progressed rapidly, and at 20 hours of life, mechanical ventilation was instituted. Chest radiograph demonstrated diffuse, bilateral interstitial infiltrates consistent with pneumonia. Persistent pulmonary hypertension was diagnosed by echocardiography. Peripheral blood culture yielded *G. adiacens* (API 20 STREP, bioMérieux Clinical Diagnostics, Durham, NC, USA) that was sensitive to vancomycin. Repeat blood samples were obtained before and after antimicrobial drug treatment was changed to vancomycin, 10 mg/kg every 12 h, and gentamicin, 4 mg/kg every 24 h. CSF culture and repeat blood cultures had no growth. Vancomycin and gentamicin were administered for 14 days. The patient eventually recovered and was discharged after 25 days of hospitalization. The biochemical identification of *G. adiacens* in the blood culture was confirmed by 16S rRNA gene sequencing.

Cervical samples for culturing were obtained from the mother 4 days after delivery. After samples were plated on blood and chocolate agar, colonies were harvested from the primary plates, and pooled bacterial DNA was isolated for further analyses. A primer pair was designed to amplify a 422-bp fragment unique to *G. adiacens*. These primers (sense, 5′-GGTTTATCCTTAGAAAGGAGGT-3′, and antisense, 5′-GAGCATTCGGTTGGGCACTCTAG-3′) were used to amplify *G. adiacens* from DNA prepared from the bacterial pools isolated from maternal cervix and, as control, from DNA prepared from the infant’s blood culture isolate. DNA amplicons underwent agarose gel electrophoresis with ethidium bromide staining and were visualized under ultraviolet light. PCR amplification showed DNA amplicons for the patient’s blood culture, his mother’s cervical swab sample, and a positive control but not for negative controls (Figure). Capillary nucleotide DNA sequence analysis confirmed all 3 amplicons as *G. adiacens* (not shown).

**Figure:**
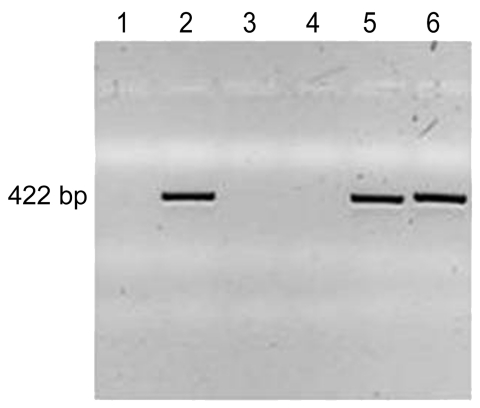
PCR amplification of *Granulicatella adiacens* DNA. DNA was prepared from the bacteria indicated, PCR-amplified with *G. adiacens*–specific primers, and subjected to agarose gel electrophoresis with ethidium bromide staining and ultraviolet light visualization. Lane 1, water, negative control; lane 2, *G. adiacens*–positive control, ATCC 49175; lane 3, DH5α *Escherichia coli*–negative control (Invitrogen, Carlsbad, CA, USA) 18263–012; lane 4, *Enterobacter sakazakii*–negative control, ATCC BAA-894 (American Type Culture Collection, Manassas, VA, USA); lane 5, patient’s blood culture isolate; lane 6, DNA isolated from a pool of bacteria obtained from the cervix of the patient’s mother. DNA amplicons were amplified only from the positive control, the patient’s blood culture, and his mother’s cervical swab specimen. Sequence analysis confirmed all 3 amplicons as *G. adiacens.*

Nutritionally variant streptococci have been difficult to identify because they have unique culture requirements, they exhibit pleomorphic phenotypic features (*1*), and they demonstrate satellitism around colonies of other bacteria. This has led to misidentification or nonidentification by using commercially available phenotypic testing, prompting the recommendation that 16S rRNA gene sequencing be used for *G. adiacens* identification (*6*).

The Clinical and Laboratory Standards Institute does not recommend disk diffusion testing to determine the susceptibility of *G. adiacens* to antimicrobial drugs and suggests broth microdilution MIC testing in cation-adjusted Mueller-Hinton broth with 2.5%–5% lysed horse blood and 0.001% pyridoxine hydrochloride (*7**,**8*). Under these conditions, 55% of *G. adiacens* isolates were susceptible to penicillin, 63% were susceptible to ceftriaxone, 96% were susceptible to meropenem, and 100% were susceptible to vancomycin (*4*). A more recent report indicates that the resistance of *G. adiacens* to β-lactam and macrolide antimicrobial drugs may be increasing (*3*).

Treatment of *G. adiacens* infection may be difficult. In vitro antimicrobial drug susceptibility patterns do not correlate well with clinical response to treatment, and *G. adiacens* infections often respond poorly to antimicrobial drug treatment. Higher rates of bacteriologic failure and relapse rates after treatment have been observed for *G. adiacens* infection than for infections with streptococci and other related genera (*9*). *G. adiacens* endocarditis has been associated with a more severe clinical course than endocarditis caused by enterococci or viridans group streptococci, with higher rates of illness and death. On the basis of studies demonstrating synergy in vitro and in vivo, empiric combination therapy with vancomycin and an aminoglycoside has been suggested until susceptibility data are available (*10*), particularly when treating serious conditions such as endocarditis (*3*).

Reports have increased of serious infections caused by *Granulicatella* and *Abiotrophia,* including this report of early-onset neonatal sepsis due to *G. adiacens*. One recent study described 8 isolates associated with invasive infection in a 4-year period (*3*). Increased awareness and identification of *G. adiacens* are likely contributing to this increased recognition, with earlier cases not detected or diagnosed. 16S rRNA gene sequencing may facilitate or confirm *Granulicatella* and *Abiotrophia* spp. as the etiologic agents of infection.

## References

[1] Christensen JJ, Facklam RR. *Granulicatella* and *Abiotrophia* species from human clinical specimens. J Clin Microbiol. 2001;39:3520–3. 10.1128/JCM.39.10.3520-3523.2001 11574566 PMC88382

[2] Gensheimer WG, Reddy SY, Mulconry M, Greves C. *Abiotrophia/Granulicatella* tubo-ovarian abscess in an adolescent virginal female. J Pediatr Adolesc Gynecol. 2010;23:e9–12. 10.1016/j.jpag.2009.05.007 19682931

[3] Zheng X, Freeman AF, Villafranca J, Shortridge D, Beyer J, Kabat W, Antimicrobial susceptibilities of invasive pediatric *Abiotrophia* and *Granulicatella* isolates. J Clin Microbiol. 2004;42:4323–6. 10.1128/JCM.42.9.4323-4326.2004 15365035 PMC516320

[4] Tuohy MJ, Procop GW, Washington JA. Antimicrobial susceptibility of *Abiotrophia adiacens* and *Abiotrophia defectiva.* Diagn Microbiol Infect Dis. 2000;38:189–91. 10.1016/S0732-8893(00)00194-2 11109021

[5] Bizzarro MJ, Raskind C, Baltimore RS, Gallagher PG. Seventy-five years of neonatal sepsis at Yale: 1928–2003. Pediatrics. 2005;116:595–602. 10.1542/peds.2005-0552 16140698

[6] Woo PC, Fung AM, Lau SK, Chan BY, Chiu SK, Teng JL, *Granulicatella adiacens* and *Abiotrophia defectiva* bacteraemia characterized by 16S rRNA gene sequencing. J Med Microbiol. 2003;52:137–40. 10.1099/jmm.0.04950-0 12543919

[7] Jorgensen JH, Hindler JF. New consensus guidelines from the Clinical and Laboratory Standards Institute for antimicrobial susceptibility testing of infrequently isolated or fastidious bacteria. Clin Infect Dis. 2007;44:280–6. 10.1086/510431 17173232

[8] Clinical and Laboratory Standards Institute. Methods for antimicrobial dilution and disk susceptibility testing of infrequently isolated or fastidious bacteria; approved guideline–second edition. 30: (M45–A2). Wayne (PA): The Institute; 2010.

[9] Stein DS, Nelson KE. Endocarditis due to nutritionally deficient streptococci: therapeutic dilemma. Rev Infect Dis. 1987;9:908–16. 10.1093/clinids/9.5.908 3317735

[10] Bouvet A. Human endocarditis due to nutritionally variant streptococci: *Streptococcus adjacens* and *Streptococcus defectivus.* Eur Heart J. 1995;16(Suppl B):24–7. 7671920 10.1093/eurheartj/16.suppl_b.24

